# Fatty acid profile, physicochemical composition and sensorial attributes of salted and sun-dried meat from young Nellore bulls supplemented with condensed tannins

**DOI:** 10.1371/journal.pone.0216047

**Published:** 2019-04-26

**Authors:** Susana Melo Gesteira, Ronaldo Lopes Oliveira, Jaqueline da Silva Trajano, Cláudio Vaz Di Mambro Ribeiro, Emellinne Ingrid de Sousa Costa, Rebeca Dantas Xavier Ribeiro, Elzânia Sales Pereira, Leilson Rocha Bezerra

**Affiliations:** 1 Department of Veterinary Medicine and Animal Science, Federal University of Bahia, Salvador, Bahia, Brasil; 2 Department of Animal Science, Federal University of Ceara, Fortaleza, Ceara, Brazil; 3 Department of Animal Science, Federal University of Campina Grande, Patos, Paraiba, Brazil; University of Illinois, UNITED STATES

## Abstract

The objective of this study was to evaluate the effect of condensed tannin inclusion (0, 10, 30 or 50 g/kg of dry matter (DM) total) from *Acacia mearnsii* extract on the fatty acid profile, physicochemical quality and sensorial analysis of salted and sun-dried meat from young Nellore bulls. The inclusion of condensed tannin extract in the young bulls’ diets promoted a quadratic reduction in the lipid content. There was a linear increase in the water retention capacity, cooking weight loss and C18:3*n–3* and a linear reduction in collagen, C16:0, C16:1*cis–*9, C18:1, MUFAs, and Δ9–desaturaseC18 in the salted and sun-dried meat from young Nellore bulls supplemented with condensed tannin. The myristic fatty acid (C14:0) and the flavor sensory attribute presented a quadratic increase. The inclusion of condensed tannin extract in the young Nellore bulls’ diets did not influence most of the physicochemical characteristics, fatty acids and nutraceutical compounds, including CLA, atherogenicity, thrombogenicity and the h:H index, the tenderness and the global appearance of the salted and sun-dried meat. Condensed tannins reduce ruminal biohydrogenation and improve the PUFA content of salted and sun-dried meat from young Nellore bulls.

## Introduction

Salted and sun-dried meat is a processed product that is a traditional processed product that is popular in Brazil. This product is based on the sequential application of salt, dehydration and maturation, which prevent the growth of undesirable microorganisms [1]; therefore, this method is widely used for conservation purposes [2]. Therefore, salted and sun-dried meat scavenge the active forms of reactive oxygen species involved in the initiation step or progression of oxidation, and thus maintain the overall quality of meat [3].

In addition to the proportion of PUFA in muscle membranes, the amount of reactive oxygen species produced and the levels of endogenous or nutritional antioxidants also influence the lipid oxidation process [4]. Moreover, lower percentages of total lipids and saturated fatty acids and higher mono- and polyunsaturated fatty acid (PUFA), conjugated linoleic acid (CLA), and n–3 and n–6 fatty acid contents are desirable to improve the functional meat properties by increasing the nutraceutical molecule contents [5,6].

Because ruminal biohydrogenation is the main factor that affects the fatty acid profile absorbed by ruminants, changes in the microbial ecology that affects the extent of fatty acid biohydrogenation can improve the fatty acid profile in the tissues [7]. Tannin has been shown to impair biohydrogenation [7,8]. Condensed tannins (or proanthocyanidins) are phenolic compounds found in forage legumes, trees and shrubs; the main characteristics of these compounds are the ability to form hydrogen bonds with proteins and act as antioxidants [9,10]. Condensed tannins can promote the incorporation of antioxidants into the cell membranes and protect the tissues against oxidation by reactive oxygen [3]. Condensed tannins ingested by ruminants interact with proteins present in the saliva, mucosa and ruminal microbiota and thus are normally considered antinutritive compounds. However, when consumed in balanced amounts (i.e., up to 5% of the diet DM, the *Acacia mearnsii* extract contains condensed tannin that may act as an additive for ruminants by reducing methane production and excessive ruminal protein breakdown and interfering with biohydrogenation [11,12]. Thus, an alternative is the use of tannin extracts from *Acacia mearnsii* extract [12] as a dietary additive.

Thus, the use of condensed tannins in animals' diet can promote the incomplete biohydrogenation of lipids and provide a higher concentration of PUFAs, which will consequently be incorporated into the meat by absorption into the small intestine of the animal, thereby improving the nutritional quality of the meat [13,14]. In addition, the salt present in the processing step of salted and sun-dried meat promotes protein denaturation [15] and has a pro-oxidant effect on myoglobin and fat [2]. Therefore, the objective of this study was to test the hypothesis that the inclusion of condensed tannins in the diet of young Nellore bulls improves the physical-chemical quality, fatty acid content and sensorial attributes of salted and sun-dried meat, thereby improving its quality and consumers’ acceptance.

## Materials and methods

### Ethical considerations and study location

This study was conducted at the Federal University of Bahia in strict accordance with the recommendations of the Guide for the Care and Use of Agricultural Animals in Research and Teaching and was approved by the Committee on the Ethics of Animal Experiments of the Federal University of Bahia, Bahia State, Brazil (Protocol Number 01/2015). This research was submitted and approved also by the Brazil Platform in its Ethical and Methodological aspects according to the guidelines established in Resolution 466/2012 and complementary to the National Health Council, approved by the Research Ethics Committee of the Federal University of Bahia. Before participating in the sensorial meat attributes, the participants signed the informed consent form. The responsible researcher signed the term of responsibility ensuring that the panelist identification was preserved.

### Animals and treatments

Thirty-two uncastrated, young Nellore (*Bos taurus indicus*) bulls averaging 17 months old and 357 ± 23.4 kg in body weight who were vaccinated and treated with antiparasitics (Ivermectin, São Paulo, Brazil) were used in a completely randomized design with four treatments and eight replications. The animals were supplemented with one of the following treatments: 0 (control), 10, 30 or 50 g/kg of dry matter (DM) of condensed tannin extract (*Acacia mearnsii*; Weibull AQ, TanacS, A., Montenegro, RS, Brazil) containing 720 g/kg of tannin in the extract. The extract was derived from the bark of *A*. *mearnsii* through extraction with hot water, vacuum evaporation and subsequent spray-drying.

The young bulls were individually housed in stalls (2.0 m × 4.0 m) with concrete floors and individual feeders and drinking troughs; the stalls were allocated in a partially covered area. The experimental period lasted 105 days, with 15 days of adaptation and a 90-day data collection period.

### Diets and chemical compositions

The diets were formulated to provide an average gain of 1.30 kg/day as recommended by the National Research Council (NRC) [16] with a 40:60 forage:concentrate ratio. Tifton hay (*Cynodon* sp.) was cut (5 cm) and mixed with the concentrate, which consisted of ground whole corn, soybean meal, mineral mixture, urea soybean and condensed tannin extract (Table 1). The diets were offered twice daily at 8:00 and 16:00 h with 10% refusals. Water was provided *ad libitum*.

**Table 1:** Ingredient proportions and the chemical and fatty acid (g/100 g of FAME) compositions of the experimental diets.

Variables	Condensed tannin^€^ (g/kg DM)			
	0	10	30	50
Ingredients (g/kg DM)				
Ground corn	445	433	413	388
Soybean meal	87.5	90.0	90.0	95.0
Condensed tannin extract^**€**^	-	10.0	30.0	50.0
Mineral mixture^¥^	10.0	10.0	10.0	10.0
Urea + ammonium sulfate^†^	15.0	15.0	15.0	15.0
Soybean oil	42.5	42.5	42.5	42.5
Tifton-85 hay	400	400	400	400
Chemical composition (g/kg DM)				
Dry matter (g/kg as fed)	835	837	839	842
Ash	45.4	45.7	46.2	46.8
Crude protein	152	152	151	151
Ether extract	63.6	63.2	62.6	62.0
Neutral detergent fiber ap^c^	375	374	371	367
Non-fibrous carbohydrate	391	392	397	399
Fatty acids (g/100 g FAME)				
C14:0	0.25	0.25	0.25	0.25
C16:0	20.7	20.6	20.2	19.9
C16:1 *cis–*9	0.10	0.09	0.09	0.09
C18:0	2.34	2.33	2.30	2.27
C18:1 *cis–*9	16.2	15.9	15.4	14.7
C18:2 *n–6*	33.6	33.2	32.3	31.4
C18:3 *n–3*	14.8	14.8	14.8	14.8

^**€**^Condensed tannin extract of *Acácia mearnsii* (Weibull AQ, Tanac S.A., Montenegro, RS, Brazil).

^¥^Maximum levels guaranteed (/kg of active ingredients): calcium, 145 g; phosphorus, 96.8 g; sulfur, 38.0 g; copper, 1810.0 mg; cobalt, 66.0 mg; iron, 2,846 mg; iodine, 89.5 mg; manganese, 1,774 mg; selenium, 14.9 mg; zinc, 4,298 mg; and fluoride, 968 mg.

^†^Mixture of urea and ammonium sulfate at a 9:1 ratio.

Samples of the ingredients and refusals were dried in a forced-ventilation oven at 55°C for 72 h and ground in a Wiley mill (Tecnal, Piracicaba, São Paulo, Brasil) with a 1-mm screen. Fecal samples were also ground with a 3.0-mm screen and analyzed according to the AOAC [17] methods to assess the dry matter (DM; method 967.03), ash (method 942.05), crude protein (CP; method 981.10), and ether extract (EE; method 920.29) contents.

In the analyses for the determination of neutral detergent fiber (NDF) and acid detergent fiber (ADF), the Van Soest et al. [18] method was used with the modifications proposed by Ankom (Ankom Technology Corporation, Macedon, NY, USA). The NDF residue was incinerated in an oven at 600°C for 4 h, and the protein correction was determined by subtracting the neutral detergent insoluble protein [19]. The non-fiber carbohydrates (NFCs) were calculated according to Hall [20] as follows using the NDF value corrected for ash and protein: NFC = 100 –[(CP–CP from urea + urea) + NDF +EE + Ash.

### Slaughtering and salted and sun-dried meat manufacturing

After 16 h of fasting, the animals were weighed and slaughtered. During the slaughtering procedure, the animals were stunned by electronarcosis (220 V, 1.5 A for 10 seconds; Dal Pino, Santo André, SP, Brazil). Then, the carcasses were suspended and bled from the jugular vein and carotid artery before they were skinned and eviscerated according to the guidelines of the Brazilian Department of Agriculture and Livestock (n°03 Rules/00 Brazil) for the Federal Inspection Service (SIF).

The head and feet were removed, and the carcasses were placed in a cold chamber (4°C) for 24 h. The carcasses were cut, and the *semimembranosus* muscles were used for salted and sun-dried meat manufacturing according to the method of Gouvêa and Gouvêa [21]. Briefly, preparation of the sun-dried meat consisted of cleaning the pieces, removing the chips, excess fat and connective tissue, and subsequent cutting of the meat into standard-sized pieces (approximately 3.00 kg). Then, the pieces were cut open in the middle to form a sheet with a maximum thickness of 5 cm. Parallel cuts were made every 4 cm in the direction of the fiber to facilitate penetration of the salt. Salting was performed with manual rubbing of 5% (150 g) sodium chloride (refined cooking salt) relative to the individual piece weight (3.00 kg). The meat was maintained for 16 h at room temperature (25°C) in plastic trays and turned after 8 h. After 16 h, the exudate was drained, and the sheets were washed with tap water and allowed to dry at room temperature suspended by hooks for 8 h. Thus, the total processing time was 24 h. Then, the sheets were individually vacuum-packed in polyethylene bags and aluminum foil to avoid oxidation and identified by treatment; one portion was refrigerated at 4°C for the physical-chemical, sensorial and oxidation analyses. Afterwards, the salted and sun-dried meat pieces were individually placed in labeled plastic bags and chilled at -20°C for the chemical composition and fatty acid profile analyses.

### Physicochemical analysis of the salted and sun-dried meat

Determination of the moisture, protein, ash, lipid and collagen contents of the salted and sun-dried meat was performed using near infrared spectroscopy (NIR) with the FoodScan^TM^ apparatus (FOSS Analytical A/S, Hillerod, Denmark) according to the methodology approved by the AOAC [17]{. The pH was evaluated in triplicate 24 h after slaughter using a Mettler M1120x pH meter (Testo, 205 Gerate-Set, Lenzkirch, Germany) according to the AOAC [22]. Then, an average pH was calculated for each sample and used for the statistical analysis.

The salted and sun-dried meat samples were cubed in approximately 2-g pieces and placed transversely (in relation to the direction of the fibers) on circular filter paper between two acrylic plates (12 × 12 × 1 cm) for five minutes for the determination of the water holding capacity (WHC). The results were obtained by calculating the amount of water lost and were expressed as percentages [23].

Cooking weight loss (CWL) of the salted and sun-dried meat was determined with two samples that were 2.5 × 2.5-cm thick. The weight of the samples was recorded before and after cooking. The subcutaneous fat was trimmed off, and the samples were cooked on an electric grill. A stainless-steel thermocouple (Gulterm 700, Gulton do Brazil) was placed into the geometric center of each sample to record the internal temperature. The salted and sun-dried meat samples were cooked until the internal temperature reached 71°C; then, the samples were removed from the grill (George Foreman Jumbo Grill GBZ6BW, Rio de Janeiro, Brazil), placed into a plastic bag and cooled at 10°C in an ice-water bath. Using the difference between the weights before and after cooking, the cooking weight loss of each sample was obtained and expressed in percentage. The sample were kept at equilibrated temperatures of 4°C overnight for the instrumental texture analysis conducted according to the AMSA [24] method.

The salted and sun-dried meat samples were brought to room temperature prior to the Warner–Bratzler Shear Force (WBSF) analysis. At least three cores with a 1.27-cm diameter and 2.0-cm length that were parallel to the muscle fibers were removed from each sample using a cork borer. Each core was sheared perpendicular to the fiber direction. The shear force was measured with a texture analyzer (Texture Analyzer TX-TX2, Mecmesin, NV, USA) fitted with a Warner–Bratzler type shear blade with a load of five kg*f* and a cutting speed of 20 cm/min [25].

The salted and sun-dried meat color was evaluated using a transverse cut of the muscle. The meat samples were exposed to atmospheric air for 30 min [26]. Then, the L*, a* and b* coordinates were measured at three different points in the muscle on non-overlapping zones, and an average was calculated for each coordinate per animal. These measurements were performed using a Minolta colorimeter (Konica Minolta, Chroma Meter CR 410, Tokyo, Japan) that was previously calibrated with the CIELAB system using a blank tile with illuminate D65 and 10° as the standard observation points. L* is related to lightness (L* = 0 black and 100 white), a* (redness) ranges from green (−) to red (+), and b* (yellowness) ranges from blue (−) to yellow (+). The color saturation (chroma, C*) was calculated as (a*^2^ + b*^2^)^1/2^ according to Hunt and King [27].

The quantification of thiobarbituric acid reactive substances (TBARS) was used for the determination of lipid oxidation in the salted and sun-dried meat samples (triplicate) at three time points (0, 25 and 50 days after slaughter) according to Grotto et al. [28]. Approximately 0.01-kg frozen samples were milled in a multiprocessor, and 0.2 mL of BHT (0.03%) antioxidant, 50 mL of distilled water and 1 mL of an antifoam solution (alcohol amylic acid) were added. The mixture was homogenized for 1 minute, and the contents were transferred to a 250-ml Erlenmeyer flask; then, 50 mL of 4 M HCl was added, and the mixture was heated on a heating plate at 100°C. Then, 5 mL of distillate was transferred to a glass tube, and 5 mL of 0.02 M TBARS solution was added. The mixture was placed in a boiling water bath for 35 min, cooled, and then taken to a spectrophotometer (GCMS-QP2010 SE). The samples were read at 530 nm, and the results were multiplied by 7.8 and expressed as milligrams of malonaldehyde per kilogram of meat sample.

### Fatty acid profile of the salted and sun-dried meat

The fatty acids from the experimental diets were extracted according to the method of Hara and Radin [29] and transmethylated simultaneously with hexane and a mixture of methanol/acetyl chloride (20:1 v/v). The fatty acid methyl esters (FAMEs) were determined in triplicate using a gas chromatograph (GC Finnigan Focus model, Varian, Palo Alto, CA, USA) equipped with a flame ionization detector and a capillary column (CP-Sil 88, 100 m x 0.25 mm i.d. x 0.20-μm film thickness, Varian, Palo Alto, CA, USA). Hydrogen was used as the carrier gas at a flow rate of 1.8 mL/min. The initial oven temperature program was set to 70°C for 4 min, increased by 13°C/min to 175°C and held for 27 min, increased by 40°C/min to 215°C and held for 9 min, and increased for 7°C/min to 230°C and held for 9 min for a total of 65 min per sample. The injector temperature was set at 250°C, and the detector temperature was set at 300°C.

The quantification of the methyl esters of fatty acids was based on the normalization of the area [30], the samples and the standard were injected into the chromatograph together with an internal standard (Crotonic Acid, SIGMA-ALDRICH, St. United States), and the internal standard concentration used was 25.5 mg/ L. The FAMEs were identified by a comparison of the FAME retention times with those of authentic standards (FAME Mix, C4-C24, SIGMA-ALDRICH, St. Louis, USA). To quantify the fatty acid methyl esters, a response factor was generated for each fatty acid based on the sample in the standard, and the response factor of each fatty acid was obtained. All conditions to rigor in all procedures for quantification of fatty acids were ensured: quality of standards, weighing, pipetting, calibrated and clean material, and evaporation of solution solvents and concentration of solutes among others. The experimental response factors were close to the theoretical factors [31]. The results were quantified by normalizing the areas of the methyl esters and are expressed as g 100/g fatty acids methyl esters (FAME).

The sum of the total saturated fatty acids (SFAs), monounsaturated fatty acids (MUFAs) and polyunsaturated fatty acid (PUFAs) and the MUFA:SFA, PUFA:SFA, PUFA:MUFA and n–6:n–3 ratios were calculated from the fatty acid profiles. To evaluate the nutritional quality of the lipid fraction of the meat samples, the atherogenicity index (AI) and Thrombogenic index (TI) were calculated according to the equation [32]: AI = [(C12 + 4 × C14 + C16] / (ΣMUFA + Σn–6 + Σn–3); TI = (C14 + C16 + C18) / [0.5 × ΣMUFA + 0.5 × Σn–6 + 3 × / (Σn–6)]. The hypocholesterolemic and hypercholesterolemic (h:H) fatty acid ratios were calculated as h:H = = [(ΣC18:1 cis-9, C18:2 n–6, C20:4 n–6, C18:3 n–3, C20:3n–6, C20:5 n–3, and C22:6 n–3)/ (ΣC14:0 and C16:0)] [33].

The Δ^9^-desaturase activity was estimated using two fatty acids [palmitic acid (D9C16) and stearic acid (D9C18)] as follows: D9C16 = [C16:1/ (C16:0 + C16:1)] × 100 and D9C18 = [(C18:1 cis–9) / (C18:0 + C18:1 cis–9)] × 100, respectively. The elongase activity was estimated according to Smet et a. [34] with the following equation: elongase = [(C18:0+ C18:1 cis–9)/(C16:0 + C16:1 + C18:0 + C18:1 cis–9)] × 100.

### Sensory attributes of the salted and sun-dried meat

The sensory characteristics of the salted and sun-dried meat were evaluated using a panel of 80 consumers [24]. Panelists were adult people of the animal science department, and they were 40 women and 40 men comprising an age group between 18 and 45 years old accustomed to eating lamb meat. The salted and sun-dried meat samples (30 g) were grouped by treatment, placed on an electric grill (George Foreman Grill Jumbo GBZ6BW, Rio de Janeiro, Brazil) and cooked until the geometric center of the samples reached 71°C. Fragments 2.0 cm^2^ from the muscle were cut, grouped, coded and transferred to a water bath (75°C) covered with aluminum foil to keep them heated and prevent the loss of volatile aroma compounds until the sensory analyses were conducted. No condiments were added. Water and cream-cracker type biscuits were provided to remove the aftertaste between consecutive tastings.

The tests were performed between 09:00 and 12:00 h, and the panelists were placed in individual cubicles in the sensory panel room. The sensory attributes were recorded using a hedonic scale of nine points (scores ranged from 1 to 9 as follows: 1, extremely dislike to 9, extremely like) according to the AMSA standards [24]. The consumer panelists evaluated the following attributes: appearance, flavor and tenderness.

### Statistical analysis

The data was analyzed in a completely randomized design with four treatments (0, 10, 30, and 50 g/kg of DM of condensed tannin extract) and eight replications. The statistical model used was as follows:

$$Y_{ij}=\mu +Ti+e_{ij},$$

where Y_ij_ = observed value, μ = overall mean, Ti = effect of condensed tannin extract, and e_ij_ = effect of experimental error.

Polynomial contrasts were used to determine the linear and quadratic effects of the different treatment levels. The command PROC REG in the SAS [35] software (SAS Inst. Inc., Cary, NC, USA) was used. The initial weight was used in the statistical model as a covariate when significant. *P*-values less than 0.05 were considered significant. The sensory analysis also included the LEVENE test to verify the variance homogeneity using the "HOVTEST" command.

## Results

### Physicochemical composition of the salted and sun-dried meat

The inclusion of condensed tannin extract in the young bulls’ diets promoted a quadratic reduction in the lipid content (*P* = 0.010), with the lowest inclusion level of 25.5 g/kg of DM obtained for 0.78 g/100 g of salted and sun-dried meat content (Table 2). We found a linear increase in the water retention capacity (*P* = 0.002) and cooking weight loss (*P* = 0.025) and a linear reduction for the collagen (*P* = 0.006) of the salted and sun-dried meat from young Nellore bulls.

**Table 2:** Physicochemical composition of salted and sun-dried meat from young Nelore bulls fed condensed tannin extract.

Variables	Condensed tannin^€^ (g/kg DM)				SEM^†^	*P*-value^¥^	
	0	10	30	50		Linear	Quadratic
Moisture (g/100 g sun-dried meat)	71.3	72.5	71.2	71.2	0.389	0.386	0.161
Ash (g/100 g sun-dried meat)	6.93	6.23	6.80	7.02	0.249	0.461	0.079
Protein (g/100 g sun-dried meat)	19.7	20.2	20.5	20.1	0.28	0.299	0.118
Lipids (g/100 g sun-dried meat)	1.74	0.78	0.87	1.51	0.285	0.651	0.010
Final pH	5.48	5.42	5.67	5.66	0.089	0.060	0.781
Water retention capacity (%)	80.6	81.5	84.4	85.4	1.142	0.002	0.982
Cooking weight loss (%)	19.9	20.8	16.2	14.1	2.024	0.025	0.476
Shear force (kg*f*/cm^2^) ^π^	3.38	3.09	2.85	3.14	0.187	0.281	0.138
Collagen (%)	2.14	1.93	1.88	1.85	0.065	0.006	0.185
Index coloration							
L* (lightness)	35.4	34.8	33.3	33.7	1.165	0.208	0.653
a* (redness)	20.0	20.6	19.4	17.5	0.994	0.065	0.223
b* (yellowness)	6.63	6.24	6.02	5.09	0.751	0.167	0.722
Chroma (%)	21.1	20.8	20.3	18.1	1.030	0.056	0.385
Lipid oxidation^≠^							
0 day	0.53	0.58	0.58	0.66	0.057	0.056	0.159
25 days	0.92	0.73	0.68	0.73	0.057	0.150	0.060
50 days	0.97	0.96	0.82	1.12	0.082	0.419	0.209

^**€**^Condensed tannin extract from *Acácia mearnsii* (Weibull AQ, Tanac S. A., Montenegro, RS, Brasil)

^†^Standard error of the mean

^¥^*P*-values were considered significant at 0.05

^π^Warner-Bratzler shear force

^≠^mg of malonaldehyde/kg of meat

The inclusion of condensed tannin extract in the young bulls’ diets did not influence (*P* > 0.05) the final pH and the Warner-Bratzler shear force of the salted and sun-dried meat. There was no effect (*P* > 0.05) of the inclusion of condensed tannins on the color indices (L*, a*, b*, and C*). The oxidation of meat lipids for the 3 time periods (0, 25 and 50 days) had no effect (*P* > 0.05).

### Fatty acid profiles of the salted and sun-dried meat

There was a linear reduction in the C16:0 (*P* = 0.049), C16:1 *cis–*9 (*P* = 0.046), C18: 1 (*P* <0.001) and MUFA (P = 0.005) contents, whereas C14:0 had a quadratic increase (*P* = 0.049) in the salted and sun-dried meat from young Nellore bulls supplemented with condensed tannin extract (Table 3).

**Table 3:** Fatty acid composition (g/100 g FAME) in the salted and sun-dried meat from young Nelore bulls fed condensed tannin extract.

Fatty acid (g/100 g of FAME)	Condensed tannin^€^ (g/kg DM)				SEM^†^	*P*-value^¥^	
	0	10	30	50		Linear	Quadratic
C12:0	0.07	0.08	0.09	0.09	0.009	0.069	0.537
C14:0	2.04	2.52	2.12	1.86	0.179	0.240	0.049
C16:0	22.8	22.8	22.4	21.6	0.470	0.049	0.410
C18:0	2.12	2.08	1.89	1.76	0.135	0.046	0.728
C16:1	16.5	17.7	18.6	18.9	0.963	0.067	0.682
C18:1 *cis*–9	31.9	30.1	28.8	27.1	1.067	<0.001	0.931
C18:2 *n–6*	6.72	6.55	7.91	9.66	1.193	0.067	0.426
CLA (rumenic acid+isomers)	0.43	0.49	0.45	0.40	0.048	0.524	0.254
C18:3 *n–3*	0.46	0.47	0.58	0.70	0.073	0.019	0.450
C20:4 *n–6*	1.6	1.55	1.95	2.22	0.299	0.093	0.594
C20:5 *n–3*	0.5	0.47	0.57	0.69	0.098	0.164	0.434
C22:5	0.87	0.83	0.96	1.31	0.180	0.073	0.277
C22:6	0.07	0.07	0.09	0.09	0.015	0.137	0.904
SFA	43.8	46.2	45.7	45.3	1.282	0.489	0.302
MUFA	43.5	41.7	40.0	37.9	1.315	0.005	0.904
PUFA	10.9	10.6	12.7	15.3	1.796	0.065	0.433
PUFA:SFA	0.25	0.24	0.28	0.35	0.047	0.114	0.394
*Σn-6*	1.77	1.7	2.14	2.43	0.369	0.101	0.574
Σ*n–3*	1.05	1.01	1.25	1.49	0.182	0.064	0.459
*n-6*:*n–3*	1.69	1.66	1.76	1.67	0.113	0.950	0.766
Δ^9^–desaturase (C16)	8.47	8.17	7.79	7.49	0.484	0.143	0.997
Δ^9^–desaturase (C18)	65.9	63.1	60.8	58.8	1.588	0.003	0.792
Elongase	65.9	65.2	66.2	66.4	0.640	0.425	0.510
Atherogenicity index	0.57	0.65	0.59	0.55	0.035	0.434	0.109
Thrombogenicity index	1.51	1.66	1.63	1.62	0.081	0.439	0.320
h:H index	1.70	1.56	1.68	1.80	0.091	0.280	0.154

^**€**^Condensed tannin extract from *Acácia mearnsii* (Weibull AQ, Tanac S. A., Montenegro, RS, Brasil)

^†^Standard error of the mean

^¥^*P*-values were considered significant at 0.05

Regarding the long-chain polyunsaturated fatty acid composition, the diets with condensed tannins promoted a linear increase in C18:3 n*-*3 (*P* = 0.019). An average of 1.46 g/100 g of FAMEs was not identified (Data available in https://doi.org/10.6084/m9.figshare.7913834.v1). Condensed tannin extract inclusion in the diets of the young Nellore bulls promoted a linear reduction in the enzymatic Δ^9^–desaturase activity only for C18:0 (*P* = 0.035).

The C12:0, C14:0, C16:1, C18:2 n–6, CLA (rumenic acid + isomers), C20:4 n–6, C20:5 n-3, C22:5, C22:6, PUFA, SFA, total n*-*6 and n*–*3 contents, elongase activity, atherogenicity, thrombogenicity and h:H index of the salted and sun-dried meat from the young Nellore bulls were not affected (*P* > 0,05) by the inclusion of condensed tannins in the animals’ diets.

### Sensory attributes of the salted and sun-dried meat

Inclusion of condensed tannin extract in the diets of the young Nellore bulls promoted a quadratic increase (*P* = 0.049) in flavor but did not affect the tenderness and global appearance of the salted and sun-dried meat (Table 4).

**Table 4:** Sensory attributes in the salted and sun-dried meat from young Nelore bulls fed condensed tannin extract.

Attributes*	Condensed tannin^€^ (g/kg DM)				SEM^†^	*P*-value^¥^	
	0	10	30	50		Linear	Quadratic
Flavor	7.15	7.51	7.26	6.95	0.166	0.262	0.049
Tenderness	6.77	7.24	6.79	6.93	0.200	0.972	0.410
Global appearance	6.84	7.22	6.92	6.80	0.190	0.635	0.195

*Hedonic scale sensory evaluation (1 = dislike extremely and 9 = like extremely)

^**€**^Condensed tannin extract from *Acácia mearnsii* (Weibull AQ, Tanac S. A., Montenegro, RS, Brasil)

^†^Standard error of the mean

^¥^*P*-values were considered significant at 0.05

## Discussion

### Physicochemical composition of the salted and sun-dried meat

The manufacturing process for the salted and sun-dried meat promoted protein denaturation, which caused dehydration, a decreased ether extract percentage and an increased ash content compared to *in natura* meat [2,13]. However, the inclusion of condensed tannins did not modify the moisture, mineral and protein proportions in the salted and sun-dried meat. The use of condensed tannins in the diet caused a curvilinear response in the fat deposition in the muscles. Changes in the FA proportion in the meat were not expected, since the dietary FAs did not differ among the diets. However, because PUFAs decrease lipogenic enzymes [36] and tannins increase PUFAs in ruminant muscle, reduced short and medium chain fatty acid synthesis may have been the cause of the changes in the lipid content in the sun-dried meat.

The average carcass final pH across treatments was less than 5.80 as recommended [24]. An adequate pH reduction is dependent on the muscle glycogen stores and the acidification process that occurs in meat [37]. Animals suffering from prolonged stress before slaughter may deplete the glycogen stores, thereby preventing a normal pH reduction after slaughter [6]. The normal values suggested that these animals did not suffer from stress or alterations in the muscle glycogen levels when fed tannins.

There was an increase in the WHC of the salted and sun-dried meat with the inclusion of condensed tannins. Protein denaturation caused by the addition of tannins may have occurred [15]. The WHC values are greater in the salted and sun-dried meat than in the *in natura* meat [13], because the addition of sodium chloride causes an increase in the WHC due to the formation of salt-protein complexes; however, the salt solubilizes the myofibrillar proteins as it diffuses into the meat, causing dehydration and a reduction of free water in the tissue and contributing to water loss [2].

Because the average WBSF for the salted and sun-dried meat was below 3.90 kgf/cm^2^ and averaged 3.12 *kgf/*cm^2^ across treatments, the meat was considered very soft according to Belew et al. [38]. This lower WBSF is desirable to improve muscle tenderness and occurs due to increased proteolysis caused by the use of sodium chloride (5%) during processing as discussed above. The WBSF is inversely related to lipids [39] and is directly related to the collagen content. Christensen et al. [40] studied the relationship among collagen characteristics, the lipid content and the meat texture of young bulls and found that these three parameters were related to the determination of softness in meat. There was a decrease in collagen with tannin addition; therefore, the WBSF would be expected to follow the same response. However, the higher softness expected with the decrease in collagen may have been offset by the quadratic effect of tannins on the meat lipids.

The inclusion of condensed tannins in the diets did not influence the color of the salted and sun-dried meat. However, the results showed that the salted and sun-dried meat had low lightness (L*) compared to *in natura* meat. This result can be explained by the reduction of myoglobin and therefore the color a* value. Additionally, the reduction in the tonality of yellow (b*) was due to the lower lipid content, and the reduction in the saturation index (C*), which represented the color intensity, was an indicator of the oxidative state and oxidation on the surface of the meat [5]. The salt present in the sun-dried meat is responsible for the greater oxidation of myoglobin and the transformation of oxymyoglobin, which makes the meat bright red, and metamyoglobin, which makes the meat grayish-brown; thus, the salt has a pro-oxidant effect on myoglobin [2]. The same pro-oxidant effect is induced by salt, making the product more susceptible to lipid deterioration through the activation of muscle lipoxidase [41]. These results justify the increase in the oxidation in the salted and sun-dried meat stored for 25 and 50 days.

### Fatty acid profile of the salted and sun-dried meat

One of the most pronounced effect of feeding tannins to ruminants is the impairment of biohydrogenation [7,8]. The biohydrogenation process basically involves two distinct groups of microorganisms [14]; the first group is responsible for the disappearance of linolenic and linoleic acids and the formation of CLA and vaccenic acid, whereas the second group is responsible for the hydrogenation of vaccenic acid to stearic acid [42]. This second group is composed mainly of gram-positive bacteria [43]. Due to the differences in their cell walls, most feed additives that decrease ruminal biohydrogenation are associated with a decrease in the proportion of the gram-positive to Gram-negative bacterial populations. Tannins also select against gram-positive bacteria [44]. Thus, higher percentages of 18:2, 18:3 and the biohydrogenation intermediates (CLA and trans-octadecenoic acids) are expected to reach the duodenum when tannins are fed to ruminants. Changes in the profiles of FAs absorbed in the duodenum can be detected in ruminant products. In fact, higher amounts of UFAs meat [37] are often attributed to an impairment of ruminal biohydrogenation when there is no change in the dietary FAs with a concomitant decrease in the C18:0 content.

The percentage of 18:3 in the sun-dried meat increased by approximately 52% compared to the control at the highest tannin level. Although there was an approximately 44% increase in the 18:2 n–6 concentration with the highest inclusion of tannin, this difference was not significant. A similar response was observed for the PUFAs, with a 40% increase that was not significant. These results confirm that tannins impair the ruminal BH based on the overall increase in individual UFAs with dietary tannins in this study [45]. We also speculated that the percent increase in the UFAs would have been greater with more oil in the diets. Supplemental dietary FAs may interact with tannins to further increase the amounts of 18:2 and total UFAs in meat [46].

The meat from young bulls fed the highest percentage of tannins had lower C14:0 and C16:0 concentrations with a concomitant increase in 18:3 n–3 compared to the control diet. However, the C12:0 concentration did not follow the expected response. The concentration was too low and therefore not sensitive enough to detect changes in fatty acid metabolism.

The decrease in MUFAs was associated with the decrease in the C18:1 concentration. Assuming the MUFA intake was the same among the diets, the lower C18:1 concentration in the sun-dried meat was a direct response from both the lower C18:0 concentration in the tissues and the lower Δ^9^-desaturase activity. High PUFA and CLA contents may inhibit the gene expression of lipogenic enzymes [36], including fatty acid synthase and Δ^9^-desaturase [36]. Additionally, the estereospecificity of FAs for the glycerol moiety in triglyceride molecules may play a role in fatty acid profiles in ruminant tissues [4].

Nutraceutical compounds, such as CLA, the UFA:SFA and PUFA:SFA ratios, the n–3 and n–6 concentrations, the hypocholesterolemic:hypercholesterolemia ratio and the atherogenicity index (AI) of the salted and sun-dried meat did not change with the addition of condensed tannins to the young bulls’ diets. Although we observed an increase in C18:3 and a decrease in the C18:0 and C16:0 contents in the sun-dried meat, the magnitude of the changes was very low. For instance, C18:3 increased from 0.46 to 0.70 g/100 g of total fat, and C16:0 decreased from 22.8 to 21.6 g/100 g. Decreasing the SFA improves the profile of fatty acids beneficial for human health [4].

### Sensory attributes of the salted and sun-dried meat

The reduction in the meat lipid content caused by the inclusion of tannins to the diets did not alter the shear force or affect the softness of the meat. The consumer panel for the sensory analysis showed that tannins had a quadratic effect on the sun-dried meat. The lipid content in meat is associated with the texture and flavor [25,47]. The effect on the flavor of the sun-dried meat may have been caused by changes in the lipid content when the young bulls were fed condensed tannins. However, the tenderness was not significantly affected. In the present study, the scores across all attributes varied from 6.79 to 7.51. Similar results were found by Gouvêa et al. [2], who evaluated salted sun-dried meat with the inclusion of licuri cake and observed scores between 6.51 and 7.36.

## Conclusion

The inclusion of condensed tannin extract in the diets of young Nellore bulls resulted in a reduction in the lipid content and cooking weight loss. However, the inclusion did not affect the color, softness and oxidation parameters. Additionally, the inclusion of condensed tannins promoted a trend in an increase in the PUFAs sum, which improved the nutritional quality of the salted and sun-dried meat, because these precursors of the *n–3* and *n–6* family are essential for human health. Although it did not improve the overall appearance, the salted and sun-dried meat with the inclusion of condensed tannins was considered the best tasting by consumers.
