# Correction: Fatty acid profile, physicochemical composition and sensorial attributes of salted and sun-dried meat from young Nellore bulls supplemented with condensed tannins

**DOI:** 10.1371/journal.pone.0219581

**Published:** 2019-07-05

**Authors:** Susana Melo Gesteira, Ronaldo Lopes Oliveira, Jaqueline da Silva Trajano, Cláudio Vaz Di Mambro Ribeiro, Emellinne Ingrid de Sousa Costa, Rebeca Dantas Xavier Ribeiro, Elzânia Sales Pereira, Leilson Rocha Bezerra

In the Author Contributions section, Ronaldo Lopes Oliveira (RLO) should be listed as responsible for conceptualization rather than Elzânia Sales Pereira (ESP).
